# Multilevel predictors of climate change beliefs in Africa

**DOI:** 10.1371/journal.pone.0266387

**Published:** 2022-04-05

**Authors:** Juan B. González, Alfonso Sánchez

**Affiliations:** 1 Paris School of Economics and University Paris 1 Panthéon-Sorbonne, Paris, France; 2 Department of International Studies, Universidad Loyola, Dos Hermanas, Spain; University of Connecticut, UNITED STATES

## Abstract

Although Africa is the most vulnerable region to climate change, little research has focused on how climate change is perceived by Africans. Using random forest methodology, we analyze survey and climate data from second-order political boundaries to explore what predicts climate change beliefs in Africa. We include five different dimensions of climate change beliefs: climate change awareness, belief in anthropogenic climate change, risk perception, the need to stop climate change, and self-efficacy. Based on these criteria we identify five key results: (1) climate change in Africa is largely perceived through its negative impacts on agriculture; (2) actual changes in local climate conditions are related to climate change beliefs; (3) authoritarian and intolerant ideologies are associated to less climate change awareness, and a diminished risk perception and belief that it must be stopped; (4) women are less likely to be aware of climate change, and (5) not speaking French, English or Portuguese is linked to a hindered understanding of climate beliefs. Our combined results can help policy makers better understand the need to jointly consider the multilevel complexities of individual beliefs and hydroclimatic data for the development of more accurate adaptation and mitigation strategies to combat the impacts of climate change in Africa.

## Introduction

The most up-to-date scientific evidence indicates that climate change has already caused “widespread and pervasive impacts to people and ecosystems” and that it continues to be “a threat to human well-being and planetary health” [1]. Urgent mitigation and adaptation strategies are required at all levels of analysis to lessen the impact from climate change. However, such mitigation strategies are not being implemented fast enough [1]. Aside from material, institutional, and political constraints, there are cognitive barriers about climate change that also delay the implementation of these strategies [2–4]. For instance, despite strong scientific consensus about the existence of anthropogenic climate change only 51% of Africans believe that human activity is the main cause behind climate change [5]. These gaps in climate change beliefs erode public support towards environmental policies and limit individual behavioral changes that could reduce greenhouse gas emissions [6–8]. Therefore, understanding what predicts *climate change beliefs* (CCBs) is crucial for policy makers to implement effective adaptation and mitigation strategies [4, 9], especially in Africa—given current projections as the continent that will be most affected by climate change [1]. Despite of this, little research has focused on what predicts climate change beliefs in Africa. Recent metanalyses suggest that fewer than 5% of published articles included African countries in their sample [4, 8]. As CCBs and its predictors vary widely across regions [10, 11], the applicability of non-African research is, at least, questionable. This study aims to narrow this gap. We make three major contributions to the existing literature. First, we offer a specific representation of what predicts African citizens’ climate change beliefs by using disaggregated data across second-order political boundaries within 33 countries. Second, we employ a novel machine learning methodology within the social sciences (random forest) to precisely classify the predictive power of a diverse number of variables over climate change beliefs. Finally, we take a holistic approach that incorporates most of the previous predictors identified largely in Western countries to examine whether they hold true in Africa.

The remainder of this article is structured as follows. First, we briefly review the current literature on the factors that shape climate change beliefs. We then explain our data collection and operationalization process, as well as the specification of our random forest models. Finally, we present our results and discuss the main findings to offer clear patterns of what predicts climate change beliefs in Africa.

### What shapes climate change beliefs

There is widespread recognition that climate change is real and that its observable consequences can already be appreciated—such as more frequent floods and droughts in Eastern Africa, extreme precipitation trends in Central Europe and dryer conditions that increase wildfires in North America [12, 13]. Nonetheless, anthropologic climate science denial persists among the public because individuals lack sufficient information, have a poor understanding of the matter, or because they associate climate science with conspiracy theories, and the like. Recent survey data from Africa suggests that only 56% of the continent’s population have heard about climate change and about 20% believe that ordinary citizens can do nothing to stop climate change [5]. What shapes these climate change beliefs? A growing number of interdisciplinary studies suggest four possible answers. First, despite the growing availability of climate change information, much of it remains inaccessible for many and those who do have access to it are unable to understand the complex science behind such phenomenon. Second, despite having access to climate change information, people’s religious beliefs or political ideology can lead to a clash of ideas that can often result in a biased interpretation of facts. Third, scientific information about climate change is too abstract, leaving people to interpret climate change trough their own personal experience with local climate conditions. Finally, people have a “finite pool of worry”—more urgent concerns than climate change—pushing climate change concerns to the backburner.

#### Barriers to climate change information

A first argument suggests that individuals do not form correct beliefs about climate change because they face barriers to access climate information. For instance, not having a cellphone or access to the internet are barriers that limit knowledge transference about climate change, reducing the amount of information that can improve CCBs [10, 11]. A similar argument suggests that such technological barriers limit access to social media platforms, which can also provide knowledge about climate change-related debates among the public [14]. Nonetheless, these platforms can also serve as echo chambers where previous beliefs are not contrasted, but continuously reinforced [15]. A second argument shifts to educational barriers. Lack of access to education leads to less knowledge, including a detailed understanding of the climate cycle and how humans negatively impact it—or so the argument goes. In this line, in-depth case study evidence suggests that even when climate change information is available, a less educated public may lack the tools to understand it, inducing incorrect beliefs [16]. This is further corroborated by large*-N* studies that find a statistical relationship between higher levels of education and more accurate climate change beliefs [8, 10, 11, 17, 18].

#### Biased interpretation

By contrast, others suggest that even when information is attainable, people’s political and religious beliefs often clash with climate science facts, leading to a biased interpretation of climate facts in order to reconcile them with conflicting beliefs and thus reduce cognitive dissonance [19]. This phenomenon, known as motivated reasoning, has been shown to influence individual CCBs, mainly for political and religious motivations [3, 8, 20]. For instance, Hart and Nisbet [21] conducted an experiment where they presented Republican and Democratic voters in the United States with the same news story about possible climate change-related impacts on human health. Their study found that the impact of this information was interpreted differently along party lines: risk perception and support for green policies increased among Democrats, while the information produced a “boomerang effect” by reinforcing skeptical views among Republicans. Therefore, even when presented with the same information, this can be interpreted biasedly to avoid compromising political beliefs. Also, what people consider to be credible information vary depending on ideological proximity to the source, and other political variables, such as participation, trust in institutions and political perceptions also shape climate change beliefs [6, 22–24].

Religious beliefs are also important to CCBs formation, since they can make individuals interpret climate change facts in a way that avoids conflict with their beliefs. In Africa, some people attribute climate-related disasters such as droughts and floods to Allah (“Allah brings the rain. The one who causes the drought is Allah.”), Yahweh (“We gather in church and pray for rain. There is nothing we can do.”), or local deities (“whose anger can unleash flooding and destruction”) [16]. Therefore, individuals who believe in a deity are more likely to attribute climate change and its repercussions to that deity’s whim rather than to human activity [25]. Similarly, attending religious services has been linked to more incorrect CCBs [26]. However, these effects vary across religions [27, 28]. Thus, even when people have access to rigorous information and the ability to understand it, politically or religiously motivated reasoning can lead to incorrect beliefs.

#### Understanding climate change trough personal experiences

A second psychological approach suggests that individuals perceive climate change as a distant phenomenon that is more likely to affect people living elsewhere and in the distant future [29]. This psychological distance from possible climate change risks often results in a lack of emotional responses to it [30, 31]. As a consequence, individuals try to make sense of the changing natural world around them using more available and emotionally salient cues, such as local climate shocks or extreme weather events and their consequences [29]. This so-called attribute substitution suggests that personal experiences often replace science-based evidence and climate change facts [3, 32]. Previous literature has found that individuals who experience extreme climate-related events such as hurricanes, floods, or temperature anomalies tend to perceive climate change as a greater risk [26, 33–38]. Even less extreme events, such as a hotter-than-usual day, can make people more aware of and concerned about climate change [39], and increase donations to environmental charities [40]. In other words, experiencing local climate shocks or extreme weather events can construct or reinforce climate change beliefs.

#### Climate change facts take a backseat

A final argument postulates that individuals have more urgent daily concerns (e.g., “bread and butter” issues), which relegate climate science information and its possible consequences to the backburner [10, 11, 41–43]. In other words, CCBs are deemed less important than immediate day-to-day concerns. This does not suggest that people do not care about climate change and its consequences, but rather, they are seen as distant occurrences that can be dealt with when they disrupt or exacerbate more pressing matters [44].

#### Demographics

Aside from the four previously discussed pathways that account for the climate change information and beliefs linkages, previous research also finds that gender, age, and race can play a role in shaping CCBs—yet a lack of consensus remains within the literature. When it comes to gender, some studies find that females are less aware of climate change than males but have higher risk perception [16, 18, 30, 45], while others find no statistical relationship between gender and climate change beliefs [8, 11, 46, 47]. Some studies have suggested that differences in the access to climate information could explain this difference [48]. Regarding age, many studies find that young people have more accurate climate change beliefs, however, this relationship is opposite in rural areas, where young rural dwellers are less aware of climate change [8]. Arguably, in rural areas agricultural experience gained with time provides knowledge about changes in the climate cycle [41, 49]. Finally, race only appears to have a consistent influence on climate change beliefs in North America, where non-white individuals show more concern and a higher risk perception than whites, a phenomenon known as “white-male effect” [7, 45, 50]. Nevertheless, this effect is not generally supported by cross-country evidence [8]. In conclusion, evidence about demographic effects on CCBs remains elusive, and these effects seem largely eclipsed by the aforementioned information approaches [8, 18].

The discussion above identifies multiple factors that shape individual climate change beliefs. However, little research has included African countries in their analysis [10, 11, 49]. Those that do often include a limited number of predictors, which in turn, limit the understanding of what predicts CCBs in the African continent. This study aims to fill these gaps within the literature.

## Research design

### Methods

We analyse what predicts climate change beliefs in Africa using a random forest approach [51]. Random forest is a machine-learning approach that uses non-parametric recursive partitioning to produce models with high predictive accuracy [10, 52]. It can handle high-dimensional (large number of predictors) multilevel datasets with high-level interactions and non-linear relations [53], making it ideal for our dataset. For each dependent variable, we grow a classification random forest composed of 1,000 trees with a minimum node size of five. We use the *ranger* package in R, which is particularly well suited for high-dimensional data and offers a fast implementation that minimizes computational burden [54]. Its optimized computation and parallelized processing are most suitable for our high-dimensional and extensive dataset. To the best of our knowledge, this is the first study to jointly combine survey and climate data at the second administrative level to explore what predicts climate change beliefs across 33 African countries.

Random forest models are not easily interpretable on their own. To interpret them, we use some additional measures. First, we compute the variable importance measure, which ranks predictors by their predictive power (including direct and indirect effects on the dependent variable). We use the corrected Gini method to do so, because it shows no bias towards predictors with more classes, in contrast to the impurity importance, at a similar computational cost [55]. This measure shows which are the most important predictors of CCBs. Second, we use partial dependence plots to illustrate the magnitude and direction of the direct effects of significant predictors. Partial dependence plots work like marginal effects in logistic regression models: they predict responses for each level of the predictor while holding constant the rest of the variables. Despite its advantages, the random forest algorithm has some limitations. For instance, the stochastic processes that provide high flexibility and predictive accuracy can also introduce some variability in the output. Also, this output is somewhat more obscure than the output from other algorithms such as conditional inference trees. The code and data used for this analysis is available online.

### Data

#### Dependent variables

Data for our dependent variables are largely drawn from the Afrobarometer, which uses a clustered, stratified (by administrative units and by urban or rural location), multi-stage and area probability sampling design [5]. We particularly use data from the seventh round of surveys (R7), conducted between 2016 and 2018 and was the first time the Afrobarometer included climate change-related questions. The dataset is comprised of more than 45,000 observations from 33 African countries. This data is georeferenced at second-order political boundaries (see S1 File for a detailed variable speciation). This allows us to overlay our climate variables to the same areas where the Afrobarometer surveys were conducted.

We include five different dichotomous specifications for climate change beliefs. Our first specification, climate change *awareness*, measures whether survey respondents have heard about the existence or climate change or not. Second, we account for the respondents’ belief that *human activity* is the main cause behind the current changes befalling the world’s climate. Third, we are interested on whether respondents developed a *risk perception* from a changing climate that is making life worse in their country. Our final two specification examine respondent’s beliefs on whether climate change *needs to be stopped* and whether ordinary citizens can contribute to mitigation efforts—a variable known in the literature as *self-efficacy*.

#### Independent variables

We include 67 independent variables from the Afrobarometer. We include 7 variables to account for access to information, 33 for political ideology and participation, 4 for religion, 10 for economic conditions, 9 for demographics, and 4 for perceptions about the impact of climate change on agriculture. All variables are grounded on previous findings within the literature on climate change beliefs. Descriptive statistics, their corresponding Afrobarometer questions and coding scheme are included in the S1 File.

Additionally, we include four climate variables to assess the impact of local climate change on CCBs. Our first three variables are precipitation anomalies, and maximum and mean temperature anomalies. Data is from the Climate Research Unit (CRU TS) 4.0 dataset [56]. From this dataset we first obtain the raw 0.5° x 0.5° gridded data of precipitation, maximum temperature, and mean temperature, for each month. We then overlay the 0.5° x 0.5° grid over the second-level political boundaries where the Afrobarometer survey was conducted. We keep the mean values of the grids each administrative unit intersects with, weighted by the intersection area. Once we have monthly precipitation, maximum and mean temperature data for each administrative unit, we calculate their anomalies as the monthly deviation from the long-term (1961–1990) mean for that month, divided by the panel’s standard deviation [57]. We then annualize these anomalies by averaging the monthly anomalies of the 12 months before each respondent was surveyed. In the end, we obtain three main climate variables at second-order political boundaries: long-term anomalies of precipitation, maximum and mean temperatures for the year before the survey was conducted. These variables capture how precipitation and temperature anomalies differ in each administrative region (compared to the 1961–1990 baseline) during the previous 12 months to the survey being conducted.

To improve the robustness of our analysis, we also include a measurement of drought for each administrative unit. We employ the 3-months standardized precipitation evapotranspiration index (SPEI-3) from the SPEI 2.6 database [58]. Since the SPEI is also presented as 0.5° x 0.5° gridded data, the data extraction procedure is identical to the other climate variables. The SPEI data are already presented as anomalies from a long-term baseline. We annualize these anomalies by taking the average value of the 12 previous months from the date the survey was conducted. In conclusion, we have four different climatic anomalies variables: precipitation, mean temperature, maximum temperature, and drought. All of them capture the anomaly of the previous year to the survey with respect to the long-term historical baseline.

## Results and discussion

**Fig 1:**
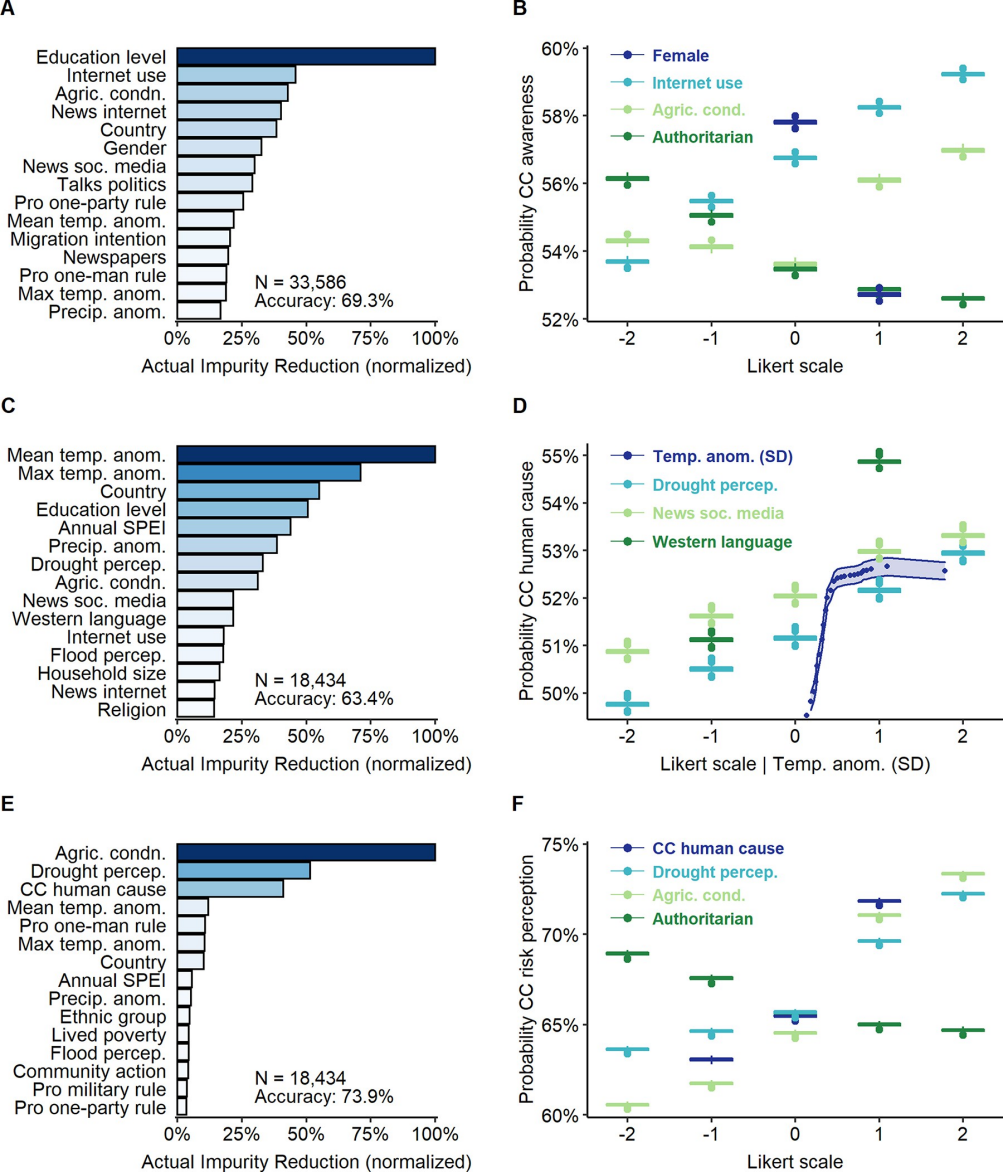
Key predictors for climate change awareness, anthropogenic climate change, and climate change-related risk perception. Top 15 predictors of climate change awareness (A) (69.4% prediction accuracy), human causation of climate change (C) (63.4% prediction accuracy), and risk perception (E) (73.9% prediction accuracy). (B) Partial dependence plot (PDP) of gender (*female*), access to online news (*news tech*), being favourable to one-party rule (*authoritarian*), and perceived agricultural conditions (*agric*. *cond*.) about climate change awareness. (D) PDP of mean temperature anomalies (*temp*. *anom*.), precipitation anomalies (*precip*. *anom*.), trust in institutions (*trust* institutions), and access to online news (*news tech*) over belief in human causation of climate change. (F) PDP of belief in human causation of CC (*CC human cause*), perceived severity of drought (*drought percep*.), perceived agricultural conditions (*agric*. *cond*.) and being favourable to one-man rule (*authoritarian*) over climate change risk perception.

In this section we present and discuss the empirical results from our random forest analysis on what predicts climate change beliefs in Africa. Some common patterns emerge among the results from our five different models. We discuss these patterns by order of statistical relevance to each outcome variable. Fig 1 shows the main results for climate change awareness, belief anthropogenic climate change, and climate change-related risk perception.

First, the importance of perceived worsening agricultural conditions stands out. Respondents’ who perceive worsening agricultural conditions show a greater awareness and perceived risk, more support for stopping climate change, and are more likely to believe in anthropogenic climate change. Indeed, Fig 1E shows that worsening perceptions of agricultural conditions are the main predictor for climate change risk perception. Arguably, the importance of the agriculture sector in terms of employment ―more than 50% of employees across Sub-Saharan Africa work in agriculture― makes agriculture a strong concern to African citizens [59]. Thus, perceiving that the impacts of climate change are bad for agriculture production may reduce the distance between climate change risks acuity and an amplified emotional response to climate-related events. These results suggest that for Africans climate change is not a problem for *others* across space and time, but rather a phenomenon that is happening *here* and *now* and with personal consequences [60]. This implies that policy makers should further highlight the global nature of climate change and its overall negative impacts on agriculture to raise CCBs, impulse individual adaptation, and mobilize public support.

**Fig 2:**
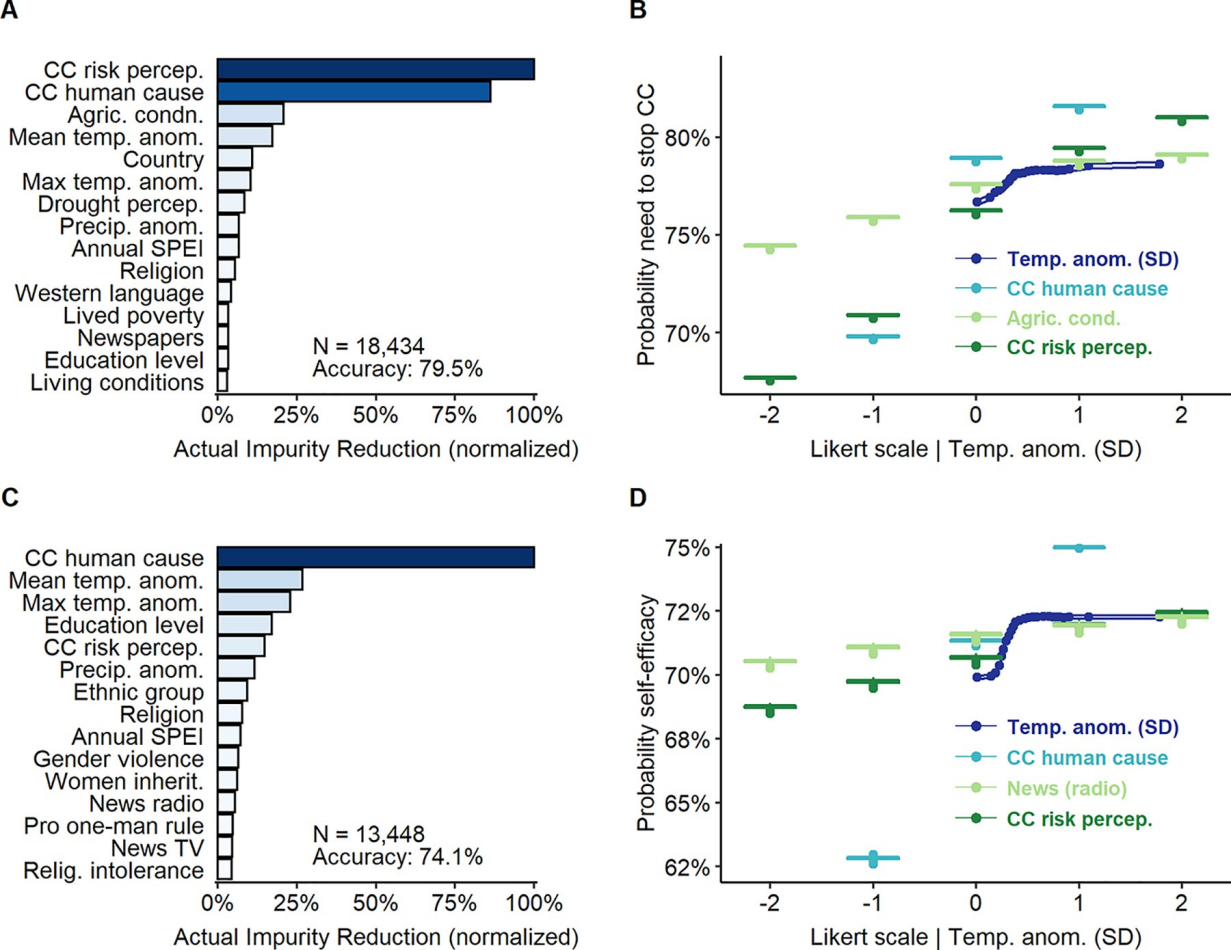
Key predictors of need to stop climate change and self-efficacy. Top 15 predictors of need to stop climate change (A) (79.5% prediction accuracy) and self-efficacy (C) (74.1% prediction accuracy). (B) Partial dependence plot (PDP) of mean temperature anomalies (*temp*. *anom*.), belief in human causation (*CC human cause*), perceived worsening of agricultural conditions (*agric*. *cond*.), and perceived risk from climate change (*CC risk percep*.) over need to stop climate change. (D) Partial dependence plot of mean temperature anomalies (*temp*. *anom*.), belief in human causation (*CC human cause*), perceived risk from climate change (*CC risk percep*.) and being favourable to one-man rule (*authoritarian*) over self-efficacy.

Second, attributing climate change to human activity is linked to higher risk perception, support for mitigation, and self-efficacy (Figs 1 and 2). If climate change is unnatural, it is extraordinary and thus riskier. Also, if climate change is human induced, its impacts can also be mitigated by human action. Besides, believing it is human caused can increase personal responsibility and, therefore, induce corrective responses [4, 61, 62]. This points to the convenience of spreading and highlighting the human origin of climate change to encourage behavioral changes and mitigation strategies in Africa.

Fig 2 shows that risk perception is positively associated with self-efficacy and the need to stop climate change. While some previous studies in the United States and United Kingdom point to fatalism or climate despair [2, 63, 64] ―where higher perceived risks discourage self-efficacy and action support, the opposite seems to be true for Africa. This could be the result of motivated control ―feeling more empowered in order to feel safe from a greater risk [65], or increased personal concern with climate change [66]. Either way, explaining why climate change is a critical risk for Africa will not discourage the African public, but it might encourage policy support and personal action [4].

As suggested by previous research, access to information and education are good predictors for believing in anthropogenic climate change and being aware of it. However, both variables show a slight negative effect when it comes to more affective dimensions such as risk perception or believing climate change must be stopped. The limited emotional salience of climatic information compared with personal experience or motivated reasoning may account for this divergence [29–31]. Nevertheless, it must be noted that the importance of information is contingent on language (Fig 1D). Not speaking French, English or Portuguese relates to a hindered understanding of climate terminology, which frequently lacks accurate translations to African languages [16]. Greater efforts should be made to translate the nature, causes, and impacts of climate change to African languages.

Local changes in climate conditions are among the most important predictors across all models. Overall, they are more important than access to news, political ideology, or demographics (see Table 1). Previous research finds that *perceived* changes in local temperatures were the most important predictor of climate risk perception in some African countries [10]. Building upon these findings, this study shows that *actual* long-term anomalies in temperature, rainfall, and drought at second-order boundaries are key predictors of individual climate change beliefs. Attribute substitution and emotional salience may explain the importance of personal experience with local weather conditions for CCBs [29, 39]. In fact, qualitative evidence suggests that many communities in Africa understand climate change not as a global phenomenon but a local one [16]. Therefore, local weather changes may be used to prime climate change and encourage mitigation and adaptation measures, but the link between those local changes and the global patterns of climate change should be highlighted.

**Table 1:** Direction of relation of key predictors to CCBs.

	Awareness	Human causation	Risk perc.	Need to stop	Self-efficacy
Agric. conditions	+	+	+	+	
Human causation			+	+	+
Risk perception				+	+
Education level	+	+		+	+
Temp. anomaly	+	+	+	+	+
Online information	+	+			
Authoritarian	-		-		-
Lived poverty			+	+	
Gender (female)	-				

Predictors ordered by overall importance across models for the top 15 predictors.

Material conditions had previously been found to influence climate change beliefs [44]. According to the “finite pool of worry" hypothesis, worse material conditions limit CCBs, as they create more urgent and pressing concerns for individuals to worry about. However, across African countries poverty has significant positive links with climate risk perception and the belief climate change must be stopped. In contrast to the finite pool of worry hypothesis, households with fewer resources are the most concerned about the present and future effects of climate change. Climate change is a close and urgent concern for them, as their income and assets are the most vulnerable to climatic risks [60, 67].

Political ideology also has a significant impact on CCBs in Africa. Authoritarian and intolerant individuals show less climate change awareness, risk perception, belief that it must be stopped and self-efficacy. Authoritarian and hierarchical values have been consistent and negatively linked to climate change beliefs in other regions of the world [8], and our results show these links also hold across Africa. Ideology influences what information people access, and how they process and assimilate it [18–21]. Our findings imply that authoritarian individuals are more likely to disregard climate change to justify their support for maintaining the status quo. These findings also suggest that it would be convenient for policy makers to reshape environmental discourses to better engage the authoritarian public. To do so, environmental policy and individual action can be framed as patriotism, innovation, or prosocial behaviour [68], and the focus of risk communication can be on the possible effects of climate change on human security and public order [69].

Like ideology, previous research has suggested that religion prompts motivated reasoning, shaping thus CCBs. Overall, declaring oneself religious is mostly insignificant to predict CCBs. However, we do observe some significant differences across religions in Africa: Catholic and Orthodox Christians are more likely to believe in the anthropogenic nature of climate change and that it must be stopped, while Sunni Muslims shows the opposite trend.

Finally, we find that demographic variables such as gender or race have some importance for predicting CCBs. We find an important gender gap for awareness, as well as slightly negative effects for other dimensions of climate change beliefs. Women are less likely to be aware of climate change, as previous case studies in Africa had suggested [16, 48, 70]. In this case, differences in access to climate information do not explain this gender gap, so further research should address this issue. Besides, we find ethnicity to be related to risk perception and self-efficacy. Overall, Black Africans show more concern and self-efficacy than other ethnic groups, among whom Arab Africans are the less concerned. Therefore, we cannot talk about a “white male” effect [50] because White Africans do not especially neglect risks (in contrast to Arab Africans) nor women show more concern, rather the opposite. Finally, age and agricultural experience are insignificant across all models, contradicting previous findings within the literature [41, 49].

Our study has three clear limitations. First, is the correlational nature of our data. Although our results offer relevant insights about the predictors of climate change beliefs in Africa, causality cannot be inferred from the available cross-sectional data. Second, random forest methodology involves a certain degree of stochasticity, which can lead to some level of randomness and uncertainty in the outcome. Finally, some variables in our dataset have less observations given that some questions within the Afrobarometer were only asked as conditioned follow-up questions.

## Conclusion

There is a lack of consensus within the literature for what predicts climate change beliefs and most studies conducted on this topic are usually limited to Western developed nations. Our results show several relevant findings for what predicts climate change beliefs across 33 African countries, using a well powered and high-dimensional dataset. First, actual changes in local climate conditions are stronger predictors of climate change beliefs in Africa than access to information, political ideology, or demographics across different model specifications. Similarly, worsening perceptions of climate conditions for agriculture is a strong predictor for all five dimensions for climate change beliefs. Second, our analysis also reveals that authoritarian and intolerant individuals show overall limited climate change beliefs. Third, not speaking French, English or Portuguese is linked to a worse understanding of climate change. Finally, there is a small but significant gender gap across different dimensions. Combined, our results suggest that more different mechanisms underly climate change beliefs than previously assumed [10]. We strongly believe that in-depth case-studies should further examine the complex causal mechanisms that shape climate change beliefs across different African countries to improve our understanding in order to implement the best mitigation and adaptation strategies for the region.

## Supporting information

S1 File Operationalization. Descriptive statistics, coding, and corresponding questions from the Afrobarometer of all variables.
(DOCX)
