# Supplementary material for: Multilevel predictors of climate change beliefs in Africa
Source: PLoS One. 2022 Apr 5;17(4):e0266387. doi: 10.1371/journal.pone.0266387 (PMC8982886; doi:10.1371/journal.pone.0266387)
Supplement: S1 File — Descriptive statistics, coding, and corresponding questions from the Afrobarometer of all variables. (DOCX) [file pone.0266387.s001.docx]

**Supporting Information 1. Operationalization**

**Table 1. Variable description**

| **Variable name** | **Afrobarometer question** | **Original answer** | **Recoded [original]** |
| --- | --- | --- | --- |
| *Climate change beliefs* |  |  |  |
| CC awareness | 73A. Have you heard about climate change or haven’t you had the chance to hear about this yet? | 0. No  1. Yes  9. Don’t Know. | 0. [0,9]  1. [1] |
| CC human cause | 74. People have different ideas about what causes climate change. What about you, which of the following do you think is the main cause of climate change, or haven't you heard enough to say? | 1. Human activity  2. Natural processes  3. Both human activity and natural processes  4. None of these  9. DK | 0. [2-9]  1. [1] |
| CC risk perception | 75. Do you think climate change is making life in [this country] better or worse, or haven’t you heard enough to say? | 1. Much better  2. Somewhat better  3. Neither / no change / about the same  4. Somewhat worse  5. Much worse  9. DK | 0. [1-3, 9]  1. [4-5] |
| Need to stop CC | 76A. Do you think climate change need to be stopped? | 0. No  1. Yes |  |
| Self-efficacy | 76B. How much do you think that ordinary citizens can do to stop climate change? | 1. Nothing at all  2. A little bit  3. A lot  9. DK | 0. [1-2, 9]  1. [3] |
| *Agricultural perceptions* |  |  |  |
| Perceived  agricultural conditions | 71. In your experience, would you say climate conditions for agricultural production in your area have gotten better, gotten worse, or stayed about the same over the last 10 years or haven’t you heard enough to say? | 0. No experience  1. Much worse  2. Worse  3. About the same  4. Better  5. Much better  9. DK | 1.  2.  3. [0, 3, 9]  4.  5. |
| Drought perception  Flooding perception | 72A. In your experience, over the past 10 years, has there been any change in the severity of the following events in the area where you live? Have they become more severe, less severe, or stayed about the same? | 1. Much more severe  2. Somewhat more severe  3. Stayed the same  4. Somewhat less severe  5. Much less severe  9. DK | Inversed |
| *Religion and ideology* |  |  |  |
| Religion | 98. What is your religion, if any? |  | 0. None / Atheist / Agnostic  1. Christian  only  2. Christian Catholic / Orthodox / Coptic  3. Christian Protestant  4. Muslim only  5. Muslim Sunni  6. Muslim Shia  7. Traditional / ethnic religion  8. Hindu  9. Other |
| Religious | 98. What is your religion, if any? |  | 0. None / Atheist / Agnostic  1. Any other |
| Religious group | 20A. Could you tell me whether you are an official leader, an active member, an inactive member, or not a member of a religious group that meets outside of regular worship services? | 0. Not a member  1. Inactive member  2. Active member  3. Official leader |  |
| Religious law | Q65. 1) Our country should be governed primarily by religious law.  2) Our country should be governed only by civil law | 1. Agree very strongly with 1  2. Agree with 1  3. Agree with neither  4. Agree with 2  5. Agree very strongly with 2 | Inversed |
| Talks politics | 13. When you get together with your friends or family, would you say you discuss political matters | 0. Never  1. Occasionally  3. Frequently |  |
| Political demonstration | 26E. Have you personally participated in a demonstration or protest march during the past year? If not, would you do this if you had the chance? | 0. Would never do this  1. Would if had the chance  2. Once or twice  3. Several times  4. Often |  |
| Democracy best | 28. Which of these three statements is closest to your own opinion? | 1. For someone like me, it doesn’t matter what kind of government we have  2. In some circumstances, a non-democratic government can be preferable  3. Democracy is preferable to any kind of government | -1. [2]  0. [3]  1. [1] |
| Democratic accountability | 29. 1) It is more important to have a government that can get things done, even if we have no influence over what it does.  2) It is more important fot citizens to be able to hold government accountable, even if that means it makes decisions more slowly. | 1. Agree very strongly with 1  2. Agree with 1  3. Agree with neither  4. Agree with 2  5. Agree very strongly with 2 |  |
| Democratic elections | 30. 1) We should choose our leaders in this country through regular, open, and honest elections.  2) Since elections sometimes produce bad results, we should adopt other methods for choosing this country’s leaders | 1. Agree very strongly with 1  2. Agree with 1  3. Agree with neither  4. Agree with 2  5. Agree very strongly with 2 | Inversed |
| Civil liberties:  - speech  - organization  - vote | 42. How often in this country do people have to be careful …  A. of what they say about politics?  B. about what political organizations they join?  C. about how they vote in an election? | 0. Never  1. Rarely  2. Often  3. Always |  |
| Full democracy | 35. In your opinion, how much of a democracy is this country today? | 1. Not a democracy  2. A democracy, but with major problems  3. A democracy, but with minor problems  4. A full democracy |  |
| Satisfaction with democracy | 36. Overall, how satisfied are you with the way democracy works in this country? | 0. This country is not a democracy  1. Not at all satisfied  2. Not very satisfied  3. Fairly satisfied  4. Very satisfied |  |
| Authoritarian:  - Pro one-party rule  - Pro military rule  - Pro one-man rule | 27. Would you disapprove or approve of the following alternatives?  A. Only one political party is allowed to stand for election and hold office  B. The army comes in to govern the country  C. Elections and Parliament are abolished so that the President can decide everything | 1. Strongly disapprove  2. Disapprove  3. Neither  4. Approve  5. Strongly approve |  |
| Free speech | 14. In this country, how free are you to say what you think? | 1. Not at all free  2. Not very free  3. Somewhat free  4. Completely free |  |
| Free media | 17. 1) The media should have the right to publish any views and ideas without government control  2) The government should have the right to prevent the media from publishing things that it considers harmful to society | 1. Agree very strongly with 1  2. Agree with 1  3. Agree with neither  4. Agree with 2  5. Agree very strongly with 2 |  |
| Free movement | 62. 1) Even if faced with threats to public security, people should be free to move about the country at any time of day or night.  2) When faced with threats to public security, the government should be able to impose curfews and set up special roadblocks to prevent people from moving around | 1. Agree very strongly with 1  2. Agree with 1  3. Agree with neither  4. Agree with 2  5. Agree very strongly with 2 | Inversed |
| Free from vigilance | 63. 1) Government should be able to monitor private communications, for example on mobile phones, to make sure that people are not plotting violence  2) People should have the right to communicate in private without a government agency reading or listening to what they are saying | 1. Agree very strongly with 1  2. Agree with 1  3. Agree with neither  4. Agree with 2  5. Agree very strongly with 2 |  |
| Gender violence | 78B. It can always be justified, is sometimes justified or is never justified for a man to beat his wife? | 1. Never justified  2. Sometimes justified  3. Always justified | Inversed |
| Women  - jobs  - land  - domestic labor | 38D. When jobs are scarce, men should have more right to a job than women  E. Women should have the same rights as men to own and inherit land?  F. In general, it is better for a family if a woman has the main responsibility for taking care of the home and children rather than a man | 1. Strongly disagree  2. Disagree  3. Neither  4. Agree  5. Strongly agree | D and F are inversed |
| Female leaders | 16. 1) Men make better political leaders than women, and should be elected rather than women.  2) Women should have the same chance of being elected to political office as men. | 1. Agree very strongly with 1  2. Agree with 1  3. Agree with neither  4. Agree with 2  5. Agree very strongly with 2 |  |
| Discriminated  Ethnic group | 85A. How often, if ever, is your Ethnic Group treated unfairly by the government? | 0. Never  1. Sometimes  2. Often  3. Always |  |
| Corruption:  - government  - Parliament  - NGOs | 44. How many of the following people do you think are involved in corruption?  A. The President and Officials in his Office  B. Members of Parliaments  J. Non-governmental organizations | 0. None  1. Some of them  2. Most of them  3. All of them |  |
| Trust President | 43A. How much do you trust the President? | 0. Not at all  1. Just a little  2. Somewhat  3. A lot |  |
| Trust Parliament | 43B. How much do you trust the Parliament? | 0. Not at all  1. Just a little  2. Somewhat  3. A lot |  |
| Intolerance:  - religious  - ethnic  - migrant | 87. For each of the following types of people, please tell me whether you would like having them as neighbors, dislike it, or not care:  A. People of a different religion  B. People from other ethnic groups  D. Immigrants | 1. Strongly dislike  2. Somewhat dislike  3. Would not care  4. Somewhat like  5. Strongly like | Inversed |
| *Material situation* |  |  |  |
| Economy past | 6. Looking back, how do you rate economic conditions in this country compared to twelve months ago? | 1. Much worse  2. Worse  3. Same  4. Better  5. Much better |  |
| Economy future | 7. Looking ahead, do you expect economic conditions in this country to be better or worse in twelve months time? | 1. Much worse  2. Worse  3. Same  4. Better  5. Much better |  |
| Living conditions | 4B. In general, how would you describe your own present living conditions? | 1. Very bad  2. Fairly bad  3. Neither good nor bad  4. Fairly good  5. Very good |  |
| Lived poverty | 8. Over the past year, how often, if ever, have you or anyone in your family:  A. Gone without enough food to eat?  B. Gone without enough clean water for home use?  C. Gone without medicines or medical treatment?  D. Gone without enough fuel to cook your food?  E. Gone without a cash income? | 0. Never  1. Just one or twice  2. Several times  3. Many times  4. Always |  |
| Perceived income quintile | 5. In general, how do you rate your living conditions compared to those of others in your country? | 1. Much worse  2. Worse  3. Same  4. Better  5. Much better |  |
| Employment | 94. Do you have a job that pays a cash income? | 0. No (not looking)  1. No (looking)  2. Yes, part time  3. Yes, full time |  |
| *Access to information* |  |  |  |
| News:  - radio  - TV  - newspapers  - internet  - social media | 12. How often do you get news from the following sources?  A. Radio  B. Television  C. Newspapers  D. Internet  E. Social media such as Facebook or Twitter | 0. Never  1. Less than once a month  2. A few times a month  3. A few times a week  4. Every day |  |
| Internet use | 91B. How often do you use the internet? | 0. Never  1. Less than once a month  2. A few times a month  3. A few times a week  4. Every day |  |
| Western language | 2B. What is the primary language you speak in your home now? |  | 0. Other  1. French, English, Portuguese |
| *Demographics and other variables* |  |  |  |
| Gender | 101. Respondent’s gender | 1. Male  2. Female | 0. [1]  1. [2] |
| Age | 1. How old are you? |  |  |
| Education level | 97. What is your highest level of education? | 0. No formal schooling  1. Informal schooling (including Koranic schooling)  2. Some primary schooling  3. Primary school completed  4. Some secondary school / high school  5. Secondary school / high school completed  6. Post-secondary qualifications, other than university  7. Some university  8. University completed  9. Post-graduate |  |
| Urban area |  | 1. Urban  2. Rural | 0. [2]  1. [1] |
| Agricultural experience | 95A. What is your main occupation or was your last main occupation?  96B. What is the main occupation of the head of the household? |  | 0. Other  1. [3. Agriculture / farming / fishing / forestry] |
| Household size | ADULT_CT. Total number of adult citizens in the household |  |  |
| Ethnic group | 102. Respondent’s race | 1. Black African  2. White / European  3. Coloured / Mixed race  4. Arab / Lebanese / North African  5. South Asian  6. East Asian  7. Other |  |
| Migration intention | 68A. How much, if at all, have you considered moving to another country to live? | 0. Not at all  1. A little bit  2. Somewhat  3. A lot |  |
| Community voluntary | 20B. Could you tell me whether you are an official leader, an active member, an inactive member, or not a member of some voluntary association or community group (not religious)? | 0. Not a member  1. Inactive member  2. Active member  3. Official leader |  |
| Community meetings | 21A. Please tell me whether you, personally, have attended a community meeting during the past year? If not, would you do this if you had the chance? | 0. Would never do this  1. Would if had the chance  2. Once or twice  3. Several times  4. Often |  |
| Community issue | 21B. Please tell me whether you, personally, have got together with others to raise an issue during the past year? If not, would you do this if you had the chance? | 0. Would never do this  1. Would if had the chance  2. Once or twice  3. Several times  4. Often |  |

**Table 2. Countries included**

| **GADM political boundaries** | **N** | **Countries** |
| --- | --- | --- |
| First-level | 3,600 | Cape Verde, Lesotho, Mauritius |
| Second-level | 42,175 | Benin, Botswana, Burkina Faso, Cameroon, Côte d’Ivoire, eSwatini, Gabon, Gambia, Ghana, Guinea, Kenya, Liberia, Madagascar, Malawi, Mali, Morocco, Mozambique, Namibia, Niger, Nigeria, São Tomé and Príncipe, Senegal, Sierra Leone, South Africa, Sudan, Tanzania, Togo, Tunisia, Uganda, Zambia, Zimbabwe |

**Table 3. Descriptive statistics**

| **Variables** | **N** | **Min** | **Max** | **Mean** | **SD** |
| --- | --- | --- | --- | --- | --- |
| *Climate change beliefs* |  |  |  |  |  |
| CC awareness | 45,732 | 0 | 1 | 0.565 | 0.496 |
| CC human causation | 26,090 | 0 | 1 | 0.507 | 0.500 |
| CC risk perception | 25,066 | 0 | 1 | 0.658 | 0.474 |
| Need to stop CC | 26,088 | 0 | 1 | 0.772 | 0.419 |
| Self-efficacy | 18,323 | 0 | 1 | 0.722 | 0.448 |
| *Agricultural perceptions* |  |  |  |  |  |
| Perceived agricultural conditions | 45,731 | -2 | 2 | 0.466 | 1.120 |
| Drought perception | 44,144 | -2 | 2 | 0.328 | 1.394 |
| Flooding perception | 44,144 | -2 | 2 | -0.209 | 1.391 |
| *Religion and ideology* |  |  |  |  |  |
| Religion | 45,775 | 1 | 10 | 4.477 | 2.816 |
| Religious | 45,775 | 0 | 1 | 0.951 | 0.216 |
| Religious group | 45,775 | -1.500 | 1.500 | -0.666 | 0.997 |
| Religious law | 44,920 | -2 | 2 | -0.548 | 1.609 |
| Talks politics | 45,775 | -1 | 1 | -0.212 | 0.711 |
| Political demonstration | 45,774 | -2 | 2 | -1.392 | 0.915 |
| Democracy best | 45,773 | -1 | 1 | 0.551 | 0.712 |
| Democratic accountability | 45,773 | -2 | 2 | 0.483 | 1.595 |
| Democratic elections | 45,773 | -2 | 2 | 0.907 | 1.482 |
| Civil liberties - speech | 45,775 | -1.500 | 1.500 | 0.464 | 1.071 |
| Civil liberties - organization | 44,358 | -1.500 | 1.500 | 0.405 | 1.103 |
| Civil liberties - vote | 45,774 | -1.500 | 1.500 | 0.489 | 1.112 |
| Full democracy | 45,690 | -1.500 | 1.500 | 0.115 | 0.916 |
| Satisfaction with democracy | 45,775 | -2.500 | 1.500 | -0.145 | 1.019 |
| Authoritarian - Pro one-party rule | 45,775 | -2 | 2 | -0.936 | 1.347 |
| Authoritarian - Pro military rule | 45,775 | -2 | 2 | -0.904 | 1.346 |
| Authoritarian - Pro one-man rule | 44,575 | -2 | 2 | -1.166 | 1.146 |
| Free speech | 45,775 | -1.500 | 1.500 | 0.435 | 1.007 |
| Free media | 45,775 | -2 | 2 | -0.062 | 1.654 |
| Free movement | 45,740 | -2 | 2 | -0.472 | 1.601 |
| Free from vigilance | 45,738 | -2 | 2 | 0.182 | 1.663 |
| Gender violence | 45,729 | -1 | 1 | 0.648 | 0.624 |
| Women - jobs | 45,775 | -2 | 2 | 0.213 | 1.544 |
| Women - land | 45,775 | -2 | 2 | 0.743 | 1.357 |
| Women - domestic labor | 45,775 | -2 | 2 | -0.256 | 1.489 |
| Female leaders | 45,775 | -2 | 2 | 0.783 | 1.556 |
| Discriminated ethnic group | 42,836 | -1.500 | 1.500 | -1.038 | 0.839 |
| Corruption - government | 45,775 | -1.500 | 1.500 | -0.111 | 0.858 |
| Corruption - Parliament | 45,775 | -1.500 | 1.500 | -0.067 | 0.811 |
| Corruption - NGOs | 45,774 | -1.500 | 1.500 | -0.313 | 0.753 |
| Trust President | 45,775 | -1.500 | 1.500 | 0.189 | 1.152 |
| Trust Parliament | 45,774 | -1.500 | 1.500 | -0.048 | 1.093 |
| Intolerance - religious | 45,716 | -2 | 2 | -0.691 | 1.218 |
| Intolerance - ethnic | 45,716 | -2 | 2 | -0.814 | 1.124 |
| Intolerance - migrants | 45,713 | -2 | 2 | -0.489 | 1.255 |
| *Material situation* |  |  |  |  |  |
| Economy past | 45,775 | -2 | 2 | -0.282 | 1.096 |
| Economy future | 45,775 | -2 | 2 | 0.289 | 1.182 |
| Living conditions | 45,770 | -2 | 2 | 0.259 | 1.248 |
| Lived poverty | 45,305 | 0 | 4 | 1.189 | 0.911 |
| Perceived income quintile | 45,775 | -2 | 2 | 0.065 | 0.970 |
| Employment | 45,568 | -1.500 | 1.500 | -0.278 | 1.178 |
| *Access to information* |  |  |  |  |  |
| News radio | 45,775 | -2 | 2 | 0.585 | 1.580 |
| News TV | 45,775 | -2 | 2 | -0.019 | 1.784 |
| Newspapers | 45,775 | -2 | 2 | -1.163 | 1.317 |
| News internet | 45,775 | -2 | 2 | -0.900 | 1.624 |
| News social media | 45,775 | -2 | 2 | -0.871 | 1.647 |
| Internet use | 45,709 | -2 | 2 | -0.726 | 1.674 |
| Western language | 45,775 | 0 | 1 | 0.115 | 0.319 |
| *Demographics and other variables* |  |  |  |  |  |
| Gender | 45,775 | 0 | 1 | 0.500 | 0.500 |
| Age | 45,729 | 18 | 106 | 37.15 | 14.94 |
| Education level | 45,496 | 0 | 9 | 3.450 | 2.238 |
| Urban area | 45,775 | 0 | 1 | 0.448 | 0.497 |
| Agricultural experience | 45,775 | 0 | 1 | 0.333 | 0.471 |
| Household size | 45,767 | 1 | 23 | 3.713 | 2.644 |
| Ethnic group | 45,775 | 1 | 6 | 1.272 | 0.862 |
| Migration intention | 45,733 | -1.500 | 1.500 | -0.702 | 1.168 |
| Community voluntary | 45,775 | -1.500 | 1.500 | -0.817 | 0.981 |
| Community meetings | 45,775 | -2 | 2 | 0.135 | 1.379 |
| Community issue | 45,774 | -2 | 2 | -0.151 | 1.349 |
